# Impact of specialty on the self-reported practice of using oral antibiotic therapy for definitive treatment of bloodstream infections

**DOI:** 10.1017/ash.2023.132

**Published:** 2023-03-09

**Authors:** Jasmine R. Marcelin, Mackenzie R. Keintz, Jihyun Ma, Trevor C. Van Schooneveld, Bryan T. Alexander, Scott J. Bergman, Molly M. Miller, Erica J. Stohs

**Affiliations:** 1 Division of Infectious Diseases, University of Nebraska Medical Center, Omaha, Nebraska; 2 Department of Biostatistics, University of Nebraska Medical Center, Omaha, Nebraska; 3 Department of Pharmaceutical and Nutrition Care, Nebraska Medicine, Omaha, Nebraska; 4 College of Pharmacy, University of Nebraska Medical Center, Omaha, Nebraska

## Abstract

**Background:**

No established guidelines exist regarding the role of oral antibiotic therapy (OAT) to treat bloodstream infections (BSIs), and practices may vary depending on clinician specialty and experience.

**Objective:**

To assess practice patterns regarding oral antibiotic use for treatment of bacteremia in infectious diseases clinicians (IDCs, including physicians and pharmacists and trainees in these groups) and non–infectious diseases clinicians (NIDCs).

**Design:**

Open-access survey.

**Participants:**

Clinicians caring for hospitalized patients receiving antibiotics.

**Methods:**

An open-access, web-based survey was distributed to clinicians at a Midwestern academic medical center using e-mail and to clinicians outside the medical center using social media. Respondents answered questions regarding confidence prescribing OAT for BSI in different scenarios. We used χ2 analysis for categorical data evaluated association between responses and demographic groups.

**Results:**

Of 282 survey responses, 82.6% of respondents were physicians, 17.4% pharmacists, and IDCs represented 69.2% of all respondents. IDCs were more likely to select routine use of OAT for BSI due to gram-negative anaerobes (84.6% vs 59.8%; P < .0001), Klebsiella spp (84.5% vs 69.0%; P < .009), Proteus spp (83.6% vs 71.3%; P < .027), and other Enterobacterales (79.5% vs 60.9%; P < .004). Our survey results revealed significant differences in selected treatment of Staphylococcus aureus syndromes. Fewer IDCs than NIDCs selected OAT to complete treatment for methicillin-resistant S. aureus (MRSA) BSI due to gluteal abscess (11.9% vs 25.6%; P = .012) and methicillin-susceptible S. aureus (MSSA) BSI due to septic arthritis (13.9% vs 20.9%; P = .219).

**Conclusions:**

Practice variation and discordance with evidence for the use of OAT for BSIs exists among IDCs versus NIDCs, highlighting opportunities for education in both clinician groups.

Bloodstream infections (BSIs) are common,^1^ and traditionally they have been managed using prolonged courses of intravenous (IV) antibiotics. Accumulating data suggests shorter courses of therapy may be appropriate in certain clinical^2^ scenarios, although debate about the route of therapy continues. Studies of gram-negative bacteremia have demonstrated successful use of shorter courses of oral fluoroquinolones for treatment of pyelonephritis with accompanying BSI,^3^ and the use of OAT for treatment of BSI due to Enterobacterales urinary tract infection (UTI) was not associated with higher failure rates and was associated with shorter hospitalizations.^4,5^ In studies of OAT for gram-positive bacteremia, *Streptococcus* spp BSI treated with OAT did not have higher rates of adverse clinical outcomes or longer hospital length of stay compared with those treated with IV therapy,^6,7^ including when β-lactams were used for OAT.^8^ Smaller studies of uncomplicated *Staphylococcus aureus* BSI have reported no difference in rates of adverse clinical outcomes with OAT as early step-down therapy (vs IV only).^9–11^ However, no randomized controlled trials have supported this yet, and IDSA guidelines currently do not recommend OAT for *S. aureus* BSI.

OAT has been used anecdotally by infectious diseases (ID) clinicians for BSI although the level of comfort with this practice vary.^12^ Concerns with using OAT for BSI may relate to bioavailability because oral β-lactam agents typically used for conditions like SSTIs may be categorized as having low bloodstream concentrations compared to dosing of their IV equivalents.^4^ In one study, however, there was no difference in mortality or risk of recurrent bacteremia when comparing oral β-lactams with trimethoprim-sulfamethoxazole (TMP-SMX) or fluoroquinolones, and these were considered a reasonable OAT option for treating Enterobacterales bacteremia from urinary sources.^13^ Without formal guidelines or strong data to support use of OAT to treat BSI, even individual ID clinician comfort with OAT for BSI varies based on experience.^14^ Because antimicrobial prescribing for BSI may occur independent of ID consultation, understanding prescribing patterns between non-ID and ID clinicians may unveil opportunities to improve patient care. We assessed self-reported OAT practice patterns among ID clinicians and non-ID clinicians.

## Methods

A web-based, open-access REDCap survey was e-mailed to clinicians caring for hospitalized patients at a large Midwestern academic medical center, including physicians, advanced practice providers, pharmacists, and trainees of each of these professions. It was also widely distributed using social media.^15^ The survey opened on October 25, 2019, by e-mail to hospital team department leaders, followed by social media launches on November 14, 2019 (Twitter) and November 19, 2019 (blog). Subsequently, 3 reminders were sent by e-mail, and the survey closed on January 31, 2020. Physicians or pharmacists who identified infectious disease as their specialty were considered infectious diseases clinicians (IDCs). All others were considered non–infectious disease clinicians (NIDCs).

We defined uncomplicated BSI cases as patients without persistent bacteremia who were clinically improving with a controlled source of infection.^16^ The survey included demographic questions and generalized treatment questions evaluating which organisms, various complicated and uncomplicated BSI syndromes, or clinical symptoms influenced respondents’ practice of using OAT to treat BSI as well as common antimicrobials they would consider using for OAT (Supplementary Material). The survey also included 6 clinical vignettes describing hospitalized patients with resolved BSIs due to a defined, pansusceptible organisms and controlled infectious syndromes: group G *Streptococcus* in cellulitis, *Escherichia coli* urinary tract infection, methicillin-resistant *S. aureus* (MRSA) gluteal abscess, *Streptococcus pneumoniae* pneumonia, methicillin-susceptible *S. aureus* (MSSA) septic arthritis, and *Prevotella oralis* ruptured appendicitis. Initial IV antibiotic therapy was described. Minimal inhibitory concentration (MIC) data for bacterial isolates were not provided.

To compare proportions of demographic factors we used χ^2^ analyses for categorical data and the Fisher exact test for analyses in which >25% of the cells had expected counts <5%. All statistical analyses were performed using SAS version 9.4 software (SAS Institute, Cary, NC) and *P* < .05 was considered significant. This survey project was judged to be exempt from approval by the Institutional Review Board of University of Nebraska Medical Center.

## Results

In total, 282 clinicians completed the survey. Among them, 233 (82.6%) were physicians, 46 (17.4%) were pharmacists, and 7 (2.5%) were advanced practice providers. Also, 195 IDCs represented 69.2% of respondents, with 87 (30.9%) NIDCs. About half of all participants (49.1%) responded via e-mail survey link, whereas 50.9% responded via social media, and 94% of respondents originated in the United States. The Twitter post received 7,831 impressions (number of times people saw the tweet) and 359 engagements, and several posts retweeted the original post about the survey. Response rate was not ascertainable due to the open-access nature of the survey and dissemination on social media.

Variability in reported practice patterns was noted between IDC and NIDC groups regarding clinical vignette responses (Table 1). Respondents reported similar practices for routine use of OAT to complete treatment, repeating of blood cultures, and duration of treatment for BSI in the clinical syndromes involving *E. coli* UTI, *S. pneumoniae* pneumonia, and streptococcal cellulitis (Table 1). With the *Prevotella* spp, BSI due to appendicitis, more IDCs reported not routinely repeating blood cultures than did NIDCs (76.7% vs 64.7%; *P* = .041). Also, more IDCs reported routinely treating this condition with ≤7 days of antibiotics than did NIDCs (57.7% vs 40.7%; *P* = .015).

**Table 1.** Use of OAT, Repeating Blood Culture, and Duration of Treatment by Clinical Vignette

Syndrome	Complete with OAT Routinely				Repeat Blood Cultures Routinely			Duration of Bacteremia Treatment			
	Yes, %	No, %	Depends, %	*P* Value	Always, %	Never, %	*P* Value	≤7 Days, %	10–14 Days, %	>14 Days, %	*P* Value
**Streptococcal BSI from cellulitis**											
IDCs (n = 195)	56.9	8.7	34.4	.781	59.5	40.5	.286	35.2	64.8	0.0	.144
NIDCs (n = 87)	60.9	6.9	32.2		52.4	47.6		31.8	65.9	2.4	
***E. coli*** **UTI with BSI**											
IDCs (n = 157)	83.5	1.6	14.9	.585	24.1	75.9	.764	59.6	40.4	0	.113
NIDCs (n = 68)	79.1	1.2	19.8		25.9	74.1		61.6	36.1	2.3	
**MRSA gluteal abscess with BSI**											
IDCs (n = 194)	11.9	60.8	27.3	**.012**	99.0	1.0	**<.0001**	1.0	72.7	26.3	**.001**
NIDCs (n = 86)	25.6	47.7	26.7		83.9	16.1		9.3	73.3	17.4	
**Pneumonia with** ***S. pneumoniae*** **BSI**											
IDCs (n = 194)	68.6	5.7	25.8	.316	47.2	52.8	.193	39.1	59.9	1.0	.163
NIDCs (n = 87)	75.9	2.3	21.8		38.4	61.6		50.0	48.8	1.2	
**Septic arthritis with MSSA BSI**											
IDCs (n = 194)	13.9	67.0	19.1	.219	98.4	1.6	**<.0001**	0.0	16.8	83.3	**<.0001**
NIDCs (n = 86)	20.9	57.0	22.1		83.1	16.9		7.0	46.5	46.5	
**Appendicitis with** ***Prevotella*** **spp BSI**											
IDCs (n = 195)	75.4%	5.1%	19.5%	.088	23.3%	76.7%	**.041**	57.7%	39.7%	2.6%	**.015**
NIDCs (n = 86)	62.8%	9.3%	27.9%		35.3%	64.7%		40.7%	52.3%	7.0%	

Note. OAT, oral antibiotic therapy; BSI, bloodstream infection; IDC, infectious diseases clinician; NIDC, noninfectious diseases clinician; UTI, urinary tract infection; MRSA, methicillin-resistant *Staphylococcus aureus*; MSSA, methicillin-susceptible *Staphylococcus aureus*. *P* values < .05 are shown in bold.

We detected differences in selected management in vignettes with infectious syndromes due to *Staphylococcus aureus*. Fewer IDCs than NIDCs selected OAT to complete treatment for MRSA BSI due to gluteal abscess (11.9% vs 25.6%; *P* = .012) and MSSA BSI due to septic arthritis (13.9% vs 20.9%; *P* = .219). The IDCs more frequently chose to always repeat blood cultures in the MRSA gluteal abscess vignette (99.0% vs 83.9%; *P* < .001) and the MSSA septic arthritis vignette (98.4% vs 93.1%; *P* < .001) than did NIDCs. Although most would treat for at least 10–14 days, more IDCs than NIDCs reported that they would treat the BSI due to MRSA gluteal abscess for >14 (17.4% vs 1.0%; *P* = .004). For MSSA BSI due to septic arthritis, more IDCs reported that they would routinely treat this bacteremia for >14 days than did NIDCs (83.3% vs 46.5%; *P* < .001). The most common OATs chosen for MRSA BSI were linezolid and TMP-SMX. IDCs were more likely to choose linezolid for OAT in this syndrome than were NIDCs (65.8% vs 26.7%), whereas NIDCs more frequently chose TMP-SMX (51.1% vs 20.6%; *P* < .001) (Table 2).

**Table 2.** Oral Antibiotics Respondents Would Consider for Treatment of Bloodstream Infection, Selected by Clinical Vignette

OAT Selected by Clinical Vignette	Fluoro- quinolone	Amoxicillin	Amoxicillin–clavulanate	Penicillin VK	Cephalexin	TMP/ SMX	Linezolid	Clinda- mycin	Azithro- mycin	Cefdinir	Cefpo- doxime	Other*
**Streptococcal BSI from cellulitis, no. (%)**												
IDCs (n = 151)	10 (6.6)	**67 (44.4)**	7 (4.6)	11 (7.3)	44 (29.1)	…	4 (2.7)	1 (0.7)	…	1 (0.7)	…	6^a^ (4.0)
NIDCs (n = 76)	…	14 (18.4)	8 (10.5)	8 (10.5)	**34 (44.7)**	4 (5.3)	1 (1.3)	2 (2.6)	…	3 (4.0)	2 (2.6)	…
***E. coli*** **UTI with BSI, no. (%)**												
IDCs (n = 176)	**93 (52.8)**	13 (7.4)	6 (3.4)	…	18 (10.2)	22 (12.5)	…	…	…	8 (4.6)	10 (5.7)	6 (3.4)
NIDCs (n = 83)	**32 (38.6)**	5 (6.0)	5 (6.0)	…	7 (8.4)	11 (13.3)	…	…	…	16 (19.3)	5 (6.0)	2 (2.4)
**MRSA gluteal abscess with BSI, no. (%)**												
IDCs (n = 73)	2 (2.7)	…	…	…	…	15 (20.6)	**48 (65.8)**	1 (1.4)	…	…	…	7 (9.6)
NIDCs (n = 45)	…	…	2 (4.4)	…	1 (2.2)	**23 (51.1)**	12 (26.7)	5 (11.1)	…	…	…	2 (4.4)
**Pneumonia with** ***S. pneumoniae*** **BSI, no. (%)**												
IDCs (n = 131)	35 (26.7)	**61 (46.6)**	6 (4.6)	9 (6.9)	1 (0.8)	…	1 (0.8)	…	1 (0.8)	7 (5.3)	7 (5.3)	3 (2.3)
NIDCs (n = 66)	15 (22.7)	8 (12.1)	**26 (39.4)**	…	1 (1.5)	1 (1.5)	…	…	3 (4.6)	9 (13.6)	2 (3.0)	1 (1.5)
**Septic arthritis with MSSA BSI, no. (%)**												
IDCs (n = 62)	1 (1.6)	1 (1.6)	2 (3.2)	…	**26 (41.9)**	10 (16.1)	7 (11.3)	1 (1.6)	…	…	…	14 (22.6)
NIDCs (n = 36)	1 (2.8)	1 (2.8)	3 (8.3)	3 (8.3)	**16 (44.4)**	1 (2.8)	2 (5.6)	4 (11.1)	…	3 (8.3)	2 (5.6)	–
**Appendicitis with** ***Prevotella*** **spp BSI, no. (%)**												
IDCs (n = 177)	16 (9.0)	…	**119 (67.2)**	1 (0.6)	…	…	…	3 (1.7)	…	1 (0.6)	…	37 (20.9)
NIDCs (n = 76)	17 (22.4)	…	**34 (44.7)**	…	1 (1.3)	1 (1.3)	…	8 (10.5)	…	3 (4.0)	…	11 (14.5)

Note. OAT, oral antibiotic therapy; BSI, bloodstream infection; IDC, infectious diseases clinician; NIDC, non-infectious diseases clinician; UTI, urinary tract infection; MRSA, methicillin-resistant *Staphylococcus aureus*; MSSA, methicillin-susceptible *Staphylococcus aureus*; TMP-SMX, trimethoprim-sulfamethoxazole. Other antibiotics written in included cefadroxil, dicloxacillin, cefuroxime, doxycycline, minocycline, (levofloxacin + rifampin combination), metronidazole, (fluoroquinolone + metronidazole combination). The most frequent consideration for each group is shown in bold.

**Fig. 1.**
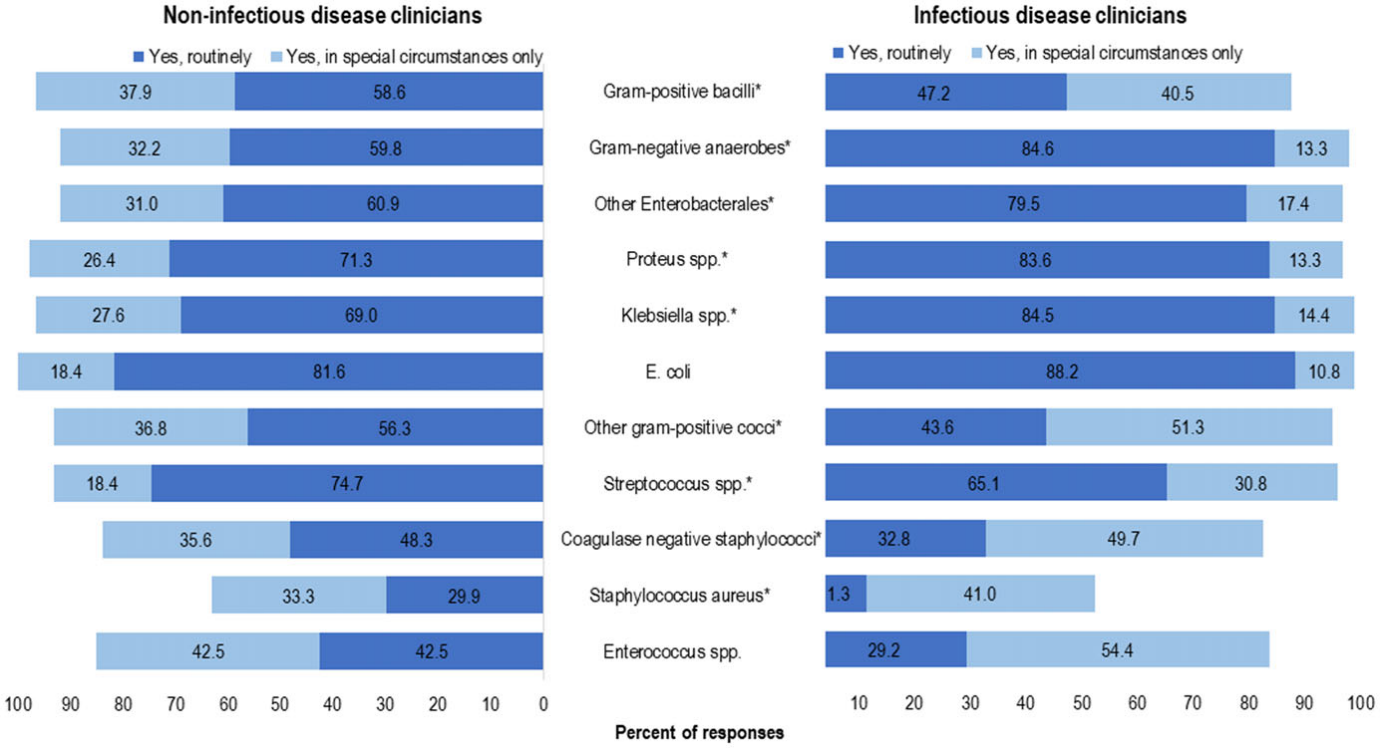
Clinician survey selection of oral antibiotic therapy to treat uncomplicated bacteremia due to specific organisms routinely or in special circumstances only. *P < .05 comparing non–infectious diseases clinicians “Yes, routinely” responses to infectious diseases clinicians’ “Yes, routinely” responses.

The antibiotics that respondents would consider for use as OAT for BSI (independent of syndrome) varied between the IDC and NIDC groups; however, >80% of respondents in both groups would consider using a fluoroquinolone, TMP-SMX, or linezolid for OAT (Table 3). The top 3 organisms that ≥80% of respondents selected OAT routinely for BSI (independent of syndrome) were *E. coli* (86.2%), *Proteus* spp (79.9%), and *Klebsiella* spp (79.7%). IDCs were more likely than NIDCs to report routine use of OAT for BSI due to gram-negative anaerobes (84.6% vs 59.8%; *P* < .0001), *Klebsiella* spp (84.5% vs 69.0%; *P* < .009), *Proteus* spp (83.6% vs 71.3%; *P* < .027), and other Enterobacterales (79.5% vs 60.9%; *P* < .004). Both IDCs and NIDCs reported routine use of OAT for BSI due to *Streptococcus* spp (65.1% vs 74.7%; *P* = .04) (Fig. 1). The reported routine use of OAT was the highest for *E. coli* in both the IDC and NIDC groups (86.2% overall; 88.2% vs 81.6% for IDS vs NIDS, respectively; *P* = .145) and the lowest for *S. aureus* (17% overall; 11.3% vs 29.9% for IDS vs NIDS, respectively; *P* = .0006).

**Table 3.** Oral Antibiotics Respondents Would Consider for Treatment of Bloodstream Infection (Independent of Clinical Syndrome)

Oral Antibiotic Regimen	Non-infectious disease clinicians (N = 87)		Infectious diseases clinicians (N = 195)		
	Would Use, No. (%)	Would Not Use, No. (%)	Would Use, No. (%)	Would Not Use, No. (%)	*P* Value^a^
Fluoroquinolone	87 (100)	0 (0)	191 (98.0)	4 (2.0)	.3152
Amoxicillin	60 (69.0)	27 (31.0)	143 (73.3)	52 (26.7)	.4747
Amoxicillin-clavulanate	80 (92.0)	7 (8.0)	147 (75.4)	48 (24.6)	**.0010**
Penicillin VK	48 (55.2)	39 (44.8)	68 (34.9)	127 (65.1)	**.0017**
Cephalexin	65 (74.7)	22 (25.3)	111 (56.9)	84 (43.1)	**.0051**
TMP-SMX	75 (86.2)	12 (13.8)	176 (90.3)	19 (9.7)	.3109
Linezolid	73 (83.9)	14 (16.1)	183 (93.9)	12 (6.1)	**.0128**
Clindamycin	64 (73.6)	23 (26.4)	98 (50.3)	97 (49.7)	**.0003**
Azithromycin	52 (59.8)	35 (40.2)	59 (30.3)	136 (69.7)	**<.0001**
Cefdinir	60 (69.0)	27 (31.0)	81 (41.7)	113 (58.3)	**<.0001**
Cefpodoxime	58 (66.7)	29 (33.3)	104 (53.6)	90 (46.4)	**.0500**
Other	5 (27.8)	13 (72.2)	15 (65.2)	8 (34.8)	**.0278**

Question prompt: “In a clinically stable hospitalized patient with bacteremia secondary to a defined source of infection, please list whether or not you would consider using the following oral antibiotics to complete therapy for bacteremia in a susceptible isolate.”

Note. TMP-SMX, trimethoprim/sulfamethoxazole.

a *P* values <.05 are shown in bold to indicate significant difference between groups selecting “would use.”

**Fig. 2.**
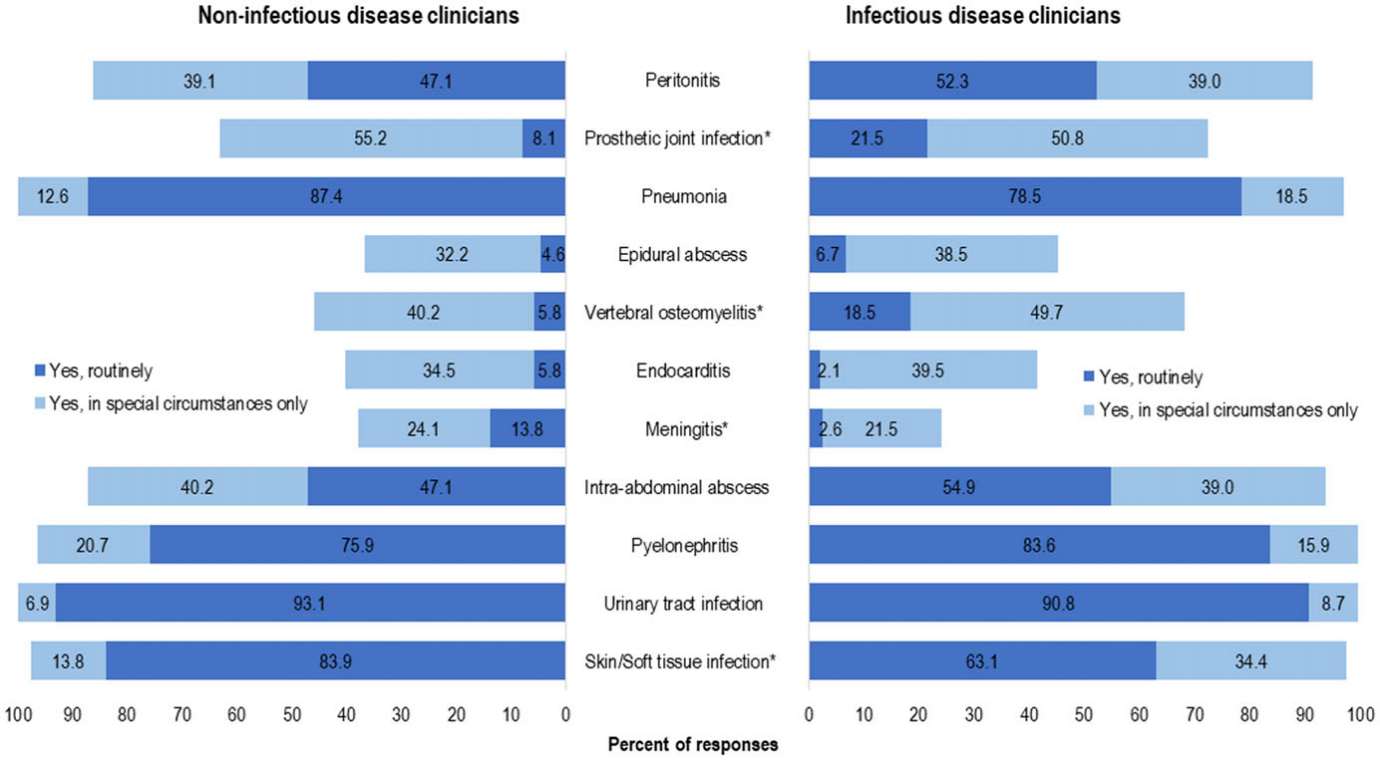
Clinician survey selection of oral antibiotic therapy to treat bacteremia due to specific syndromes routinely or in special circumstances only. *P < .05 comparing non–infectious diseases clinicians “Yes, routinely” responses to infectious diseases clinicians’ “Yes, routinely” responses.

Figure 2 includes select syndromes that may be considered complicated BSI were also included in the survey: prosthetic joint infection (PJI), vertebral osteomyelitis, endocarditis, epidural abscess, and meningitis. We detected significant differences in reported routine use of OAT for PJI by IDCs and NIDCs (42 [21.5%] of 195 IDC vs 7 [8.1%] of 87 NDICs; *P* < .05), vertebral osteomyelitis (36 [18.5%] of 195 IDCs vs 5 [5.8%] of 87 NDICs; *P* < .05), and meningitis (5 [2.6%] of 195 IDCs vs 12 [13.8%] of 87 NDICs; *P* < .05). Overall, only 81 (41.6%) of 195 IDCs would select OAT for endocarditis routinely or in special circumstances, compared with 141 (72.3%) who selected OAT in those categories for PJI and 132 (68.2%) who selected OAT in those categories for vertebral osteomyelitis.

## Discussion

In this study, we assessed the self-reported practice patterns of IDCs compared with those of NIDCs. As with most complex medical decisions, in the absence of specific guidelines outlining standard practice in each situation, respondents demonstrated varying levels of confidence in using OAT for BSI depending on the organisms or clinical syndrome being treated. More complicated clinical scenarios were associated with more discordant responses among IDCs and NIDCs. Both IDCs and NIDCs reported transition to OAT for BSI patients with *E. coli* due to pyelonephritis, group G *Streptococcus* due to cellulitis, and *S. pneumoniae* due to pneumonia. This finding is consistent with extrapolation of published data for outcomes in patients treated with OAT for the primary infections via subgroup analysis of bacteremic patients within those cohorts and correlates with results of the survey by Hospenthal et al^12^ regarding IDC practice patterns.^12^

Most data on OAT for definitive treatment of BSI involve infections with gram-negative organisms, particularly Enterobacterales, often associated with uncomplicated clinical syndromes like UTIs,^5,17^ treated with highly bioavailable oral antibiotic options.^17–19^ Early transition to OAT for treatment of Enterobacterales BSI has not resulted in increased mortality or treatment failure and may benefit patients by reducing IV-line–associated complications and hospital length of stay.^5,17,20,21^ As additional data have accumulated on this topic, a higher frequency of reported use of OAT for gram-negative BSI, especially due to certain Enterobacterales, is expected. Furthermore, the practices of both IDC and NIDC respondents were congruent with data supporting a short duration of treatment^16^ and fewer unnecessary repeat blood cultures^22^ for these organisms. This finding also aligns with national^12^ and international^23,24^ surveys of IDCs. However, our data suggest that while most clinicians are aware of the acceptability of using OAT to treat BSI due to *E. coli* as shown by the clinical vignette, it appears that additional opportunities exist for education regarding similar treatment of other Enterobacterales (eg, *Klebsiella* spp and *Proteus* spp, etc) because NIDCs were less likely to choose OAT for BSI due to those organisms than were IDCs. Studies identifying the successful use of OAT for Enterobacterales BSI included these organisms in significant quantities and demonstrated similar outcomes.^25^ Therefore, we may have identified a gap in NIDC recognition of patients with uncomplicated non–*E. coli* Enterobacterales BSI as potential good candidates for OAT. Targeted education on this topic may be beneficial.

Fewer data are available regarding use of OAT for BSI due to gram-positive bacteria,^6–8^ and what data are available typically centers around syndromes of SSTIs and pneumonia. However, a few studies have evaluated the use of OAT for *Streptococcus* spp. BSI, demonstrating shorter length of stay without increased bacteremia recurrence, mortality, or adverse effects.^6,8^ There was no discordance between IDC and NIDC confidence in using OAT for streptococci and other nonstaphylococcal gram-positive cocci. However, it was surprising that <60% of IDCs reported OAT as a definitive treatment for uncomplicated streptococcal BSI due to cellulitis; we expected this group to report more use of OAT in this syndrome. Despite recommendations against obtaining routine blood cultures in individuals with cellulitis,^26,27^ the data demonstrating that these are frequently obtained,^28^ combined with a lower-than-expected use of OAT in this syndrome by IDCs, suggests that additional opportunities exist for education of IDCs on the possible merits of adopting this practice.

Appropriate treatment of *Staphylococcus aureus* BSI is highly effective for clinical outcomes, and ID consultation has been demonstrated to improve clinical care through adherence to guidelines and reduced mortality^29–33^ Fewer IDC reported routinely using OAT for MRSA BSI secondary to drained gluteal abscess than NIDCs, and IDCs were more likely to report longer treatment and routinely repeating blood cultures. When they chose OAT, more IDCs reported use of high bioavailability treatments like linezolid.^34^ The Infectious Diseases Society of America (IDSA) guidelines specifically addressing management of *S. aureus* BSI are currently under development, but other guidelines, data, and clinical experience suggest a 4–6-week duration of treatment for complicated *S. aureus* BSI with IV antibiotics.^35,36^ However, a few, small, retrospective studies of early OAT for low-risk uncomplicated *S. aureus* BSI have not demonstrated increased complications, relapse, or death with OAT compared with IV,^9–11^ although data from a randomized controlled trial to evaluate this finding have not yet been published.^37^ This paucity of data likely influenced responses in our study, with respondents less likely to select OAT for both MRSA and MSSA BSI compared to other organisms. Notably, however, more than twice the NIDCs than IDCs respondents reported that they would routinely prescribe OAT for *S. aureus* BSI, suggesting that given the nuance around treatment of *S. aureus* BSI, there may still be additional room for collaboration between IDCs and NIDCs on treating this important infection according to the most current available evidence and established standards of care, as involvement of IDC in management of *S. aureus* BSI has improved patient outcomes.^29–33^ Furthermore, this finding highlights the impact of this gap in evidence for treatment of uncomplicated *S. aureus* BSIs and the need for additional, carefully designed studies to better define the role of oral antibiotics in this syndrome.

A growing body of data supports the use of OAT, even for complicated BSI. The POET trial demonstrated that an IV to OAT switch in infective endocarditis was noninferior to continuing parenteral antibiotics.^38^ Nevertheless, few respondents would use OAT to treat endocarditis, although just more than one-third of IDCs would consider it in certain situations. The predominance of *S. aureus* causing endocarditis in our predominantly North American respondent population may have influenced this response. Similarly, the OVIVA trial reported robust data demonstrating noninferiority of OAT compared to IV antibiotics for bone and joint infection,^39^ and more IDCs have readily embraced this use of OAT. The discordance between IDC and NIDC use of OAT for these complicated infections reflects the need for the clinical nuance brought by early ID consultation. The etiology of complicated syndromes like endocarditis may limit clinicians’ blanket selection of OAT. However, the fact that only 40% would even consider OAT in special circumstances also represents gaps in implementation and assimilation of the results of high-quality RCTs into the practice of IDC themselves. This finding highlights the need for continued work to move from dogma to evidence-based treatment for these complex clinical scenarios.^40^

Although some data support the use of OAT for BSI, we identified a difference in the reported use of OAT for BSI due to select organisms or specific infectious syndromes, depending on whether the clinician was an ID specialist or not. Our data suggest that clinicians are more likely to select OAT for BSI due to UTIs, SSTIs, and pneumonia, consistent with extrapolation of published data for outcomes in patients treated with OAT for the primary infections via subgroup analysis of bacteremic patients within those cohorts. A survey conducted by Hospenthal et al^12^ reported that most IDC respondents would be comfortable transitioning to OAT for treatment of gram-negative BSI due to urinary or gastrointestinal sources, or gram-positive BSI due to central line infections, SSTIs, or pneumonia.

In one study, IV to OAT switch for BSIs occurred in 95% of eligible episodes when an ID specialist was consulted but in only 34% of cases without ID involvement.^41^ Although there are defined syndromes for which ID consultation has been shown to have mortality benefit for hospitalized patients, teams without ID consultation services may still benefit from clinical pharmacist guidance to assist with IV to OAT switches where appropriate. There have been criteria and bundles suggested for safe transition from IV to OAT for some gram-negative BSI^42–44^; however, no published guidelines specifically address OAT use in BSI, resulting in practice variability. As with most complex medical decisions, in the absence of specific guidelines outlining standard practice in each situation, respondents reported varying levels of use of OAT for BSI depending on the organisms or clinical syndrome being treated, with more complicated clinical scenarios being associated with more discordant responses among NIDCs and IDCs.

This study had several limitations. First, we are unable to quantify nonresponse bias for this electronic survey. The lack of data from individuals who were not interested in the topic or did not have time to complete the survey due to response fatigue may have influenced the results. Second, given that this survey was widely distributed on social media, there was no way to inhibit participants with no clinical training from answering. Third, no chart review was performed to verify whether survey responses represent actual clinician practice. Fourth, the small sample size and North American predominance limits the generalization of these findings; however, this study remains unique in that it included NDICs. However, this group of NIDCs likely self-selected to include those who were more interested or knowledgeable of ID practice than the average NIDC. Therefore, true differences between IDC and NIDC use of OAT may be more disparate than our results indicate. Additionally, the survey was not followed up with qualitative assessment of why respondents would decide to use or not use OAT for each clinical scenario; a future study could include focus groups to assess themes associated with antibiotic use decisions. Furthermore, the design of the survey did not allow for a response of “would not treat at all”; it is unclear whether this inadvertently affected the distribution of responses. Finally, in the design of the survey questions, metronidazole and doxycycline were inadvertently excluded from the OAT choice selections. However, because of the “other” option, respondents who wanted to report use of those agents were able to write them in.

Considerable variation in reported use of OAT for BSIs among IDCs vs NIDCs exists, highlighting clear opportunities for education of NIDCs and collaboration with IDCs regarding appropriateness and selection of OAT for BSI. However, we also identified specific clinical scenarios in which IDCs could improve practice to align with data supporting OAT for BSI, including for streptococcal BSI and endocarditis. Understanding the reasons for variability and deviations from evidence-based practice may be helpful in creating best-practice guidelines to standardize decision making.

## Supplementary material

For supplementary material accompanying this paper visit http://doi.org/10.1017/ash.2023.132. click here to view supplementary material
