# Supplementary material for: Impact of specialty on the self-reported practice of using oral antibiotic therapy for definitive treatment of bloodstream infections
Source: Antimicrob Steward Healthc Epidemiol. 2023 Mar 9;3(1):e48. doi: 10.1017/ash.2023.132 (PMC10031584; doi:10.1017/ash.2023.132)
Supplement: Supplementary file 1 [file S2732494X23001328sup001.pdf]

Marcelin JR, et al. *Impact of Specialty on the Self-Reported Practice of using Oral Antibiotic Therapy for Definitive Treatment of Bloodstream Infections.*

Supplement - Survey questions

OAT BSI Screening

Dear Participant,

The Nebraska Medicine Antimicrobial Stewardship Program (ASP) invites you to participate in a research survey entitled: Use of Oral Antibiotic Therapy (OAT) for Definitive Treatment of Uncomplicated Bloodstream Infections (uBSIs): Opportunities for Antimicrobial Stewardship.

There are no established guidelines on the use of oral antibiotics to treat bloodstream infections. The purpose of this study is to evaluate clinician's use of oral antibiotics in bloodstream infections.

The primary physical location of this study is Nebraska Medicine, but has been expanded to include clinicians outside of Nebraska Medicine using social media/digital dissemination.

You are eligible to participate in this research study because you may be a physician or advanced practice provider caring for patients who may receive antibiotics.

Your participation in this survey is voluntary and your responses will be confidential and anonymous. There are no known risks to completing this survey. If you agree to participate, please fill out this survey, which should take less than 5 minutes to complete.

If you have already completed this survey once, please do not take it again.

Sincerely,

Dr. Jasmine Marcelin MD,
Associate Medical Director, Antimicrobial Stewardship Program
Nebraska Medicine/University of Nebraska Medical Center

---

1) 1. Do you regularly prescribe antibiotics for hospitalized patients as part of your current practice (as a physician, advanced practice provider, or pharmacist)?

- ◯ Yes
- ◯ No

OAT BSI Survey

Note: If you are participating in this survey using your mobile device, you may want to adjust the text size with the resize buttons located in the upper right corner of the screen.

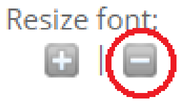

2. How did you access this survey?

○ Link sent through email ○ Social Media (Twitter, UNMC ID blog post, LinkedIn, etc.)

3. In a clinically stable, hospitalized patient with resolved bacteremia secondary to a defined source of infection, please choose which clinical conditions you would typically be comfortable using oral antibiotics to complete a course of treatment for bacteremia: (e.g. skin/soft tissue infection, endocarditis, pneumonia, etc.)

	Yes routinely	Yes, but in special circumstances only	No, never
a. Skin/Soft tissue infection	○	○	○
b. Urinary tract infection	○	○	○
c. Pyelonephritis	○	○	○
d. Intra-abdominal abscess	○	○	○
e. Meningitis	○	○	○
f. Endocarditis	○	○	○
g. Vertebral osteomyelitis	○	○	○
h. Epidural abscess	○	○	○
i. Pneumonia	○	○	○
j. Prosthetic joint infection	○	○	○
k. Peritonitis	○	○	○
l. Other (Please specify here)	○	○	○

Please specify here:

4. In a clinically stable, hospitalized patient with resolved bacteremia secondary to a defined source of infection, please choose which organisms you would feel comfortable using oral antibiotics to complete a course of treatment for bacteremia in the appropriate clinical setting:

	Yes routinely	Yes, but in special circumstances only	No, never
a. Enterococcus spp.	○	○	○
b. Staphylococcus aureus	○	○	○
c. Coagulase negative staphylococci	○	○	○
d. Streptococcus spp.	○	○	○
e. Other gram-positive cocci	○	○	○
f. E. coli	○	○	○
g. Klebsiella spp.	○	○	○
h. Proteus spp.	○	○	○
i. Other Enterobacteriaceae (enteric aerobic gram-negative)	○	○	○
j. Gram-negative anaerobes	○	○	○
k. Gram-positive bacilli (e.g. lactobacillus, corynebacterium)	○	○	○
l. Other (Please specify here)	○	○	○

Please specify here

5. In a hospitalized patient with bacteremia secondary to a defined source of infection, please list which clinical signs/symptoms you would consider as "important factors" that would influence your decision to choose oral antibiotic therapy for bacteremia:

	Important	Not important
a. Absence of fever >38.5F	○	○
b. No evidence of Sepsis	○	○
c. Improving focus of infection	○	○
d. Complete drainage of abscess	○	○
e. Absence of CNS involvement	○	○
f. Functional gastrointestinal tract	○	○
g. Able to take food or other medications by mouth	○	○
h. Other (Please specify here)	○	○

Please specify here

6. In a hospitalized patient with bacteremia secondary to a defined source of infection, please list which clinical signs/symptoms you would consider as "complicating factors" that would result in you NOT CHOOSING to use oral antibiotic therapy for bacteremia:

	Important	Not important
a. Fever >38.5F	◯	◯
b. Tachycardia	◯	◯
c. Respiratory failure	◯	◯
d. Sepsis	◯	◯
e. Slow or stalled clinical improvement of infectious focus	◯	◯
f. Undrained abscess	◯	◯
g. CNS involvement	◯	◯
h. Concern about GI tract function	◯	◯
i. Presence of diarrhea	◯	◯
j. Other (Please specify here)	◯	◯

Please specify here

7. In a clinically stable hospitalized patient with bacteremia secondary to a defined source of infection, please list whether or not you would consider using the following oral antibiotics to complete therapy for bacteremia in a susceptible isolate:

	Would use	Would not use
a. Levofloxacin/ciprofloxacin /moxifloxacin	◯	◯
b. Amoxicillin	◯	◯
c. Amoxicillin/clavulanate	◯	◯
d. Penicillin VK	◯	◯
e. Cephalexin	◯	◯
f. TMP/SMX	◯	◯
g. Linezolid	◯	◯
h. Clindamycin	◯	◯
i. Azithromycin	◯	◯
j. Cefdinir	◯	◯
k. Cefpodoxime	◯	◯
l. Other	◯	◯

Please list here

Please answer the questions following these clinical vignettes, assuming there are no other unknown conditions, and all bacterial isolates are pan-susceptible (except if explicitly stated otherwise).

8. 35-year-old male with diabetes mellitus presents with nonpurulent RLE cellulitis. He develops a fever and blood cultures are positive for group G streptococcus. He is started on IV cefazolin and improves clinically.

Would you complete this patient's antibiotic course with ORAL therapy?	◯ Yes (always)<br>◯ No (never)<br>◯ Depends (Only if no persistent fever)
Would you routinely repeat blood cultures to confirm clearance of bacteremia?	◯ Yes<br>◯ No
How long would you treat this patient's bacteremia?	◯ < 7 days<br>◯ 7 days<br>◯ 10 days<br>◯ 14 days<br>◯ > 14 days

9. 76-year-old female with presents with altered mental status and fever. She was found to have an E. coli urinary tract infection and bacteremia with the same organism. She was treated with IV ceftriaxone and improved clinically.

Would you complete this patient's antibiotic course with ORAL therapy?	◯ Yes (always)<br>◯ No (never)<br>◯ Depends (Only if no persistent fever)
Would you routinely repeat blood cultures to confirm clearance of bacteremia?	◯ Yes<br>◯ No
How long would you treat this patient's bacteremia?	◯ < 7 days<br>◯ 7 days<br>◯ 10 days<br>◯ 14 days<br>◯ > 14 days

10. A 65-year-old male with diabetes mellitus presents with fever and hypotension and was found to have methicillin-resistant S. aureus (MRSA) bacteremia, due to a left gluteal abscess that was debrided. TTE and TEE demonstrated NO evidence of endocarditis. He was treated with IV vancomycin and improved clinically.

Would you complete this patient's antibiotic course with ORAL therapy?	◯ Yes (always)<br>◯ No (never)<br>◯ Depends (Only if no persistent fever)
Would you routinely repeat blood cultures to confirm clearance of bacteremia?	◯ Yes<br>◯ No
How long would you treat this patient's bacteremia?	◯ < 7 days<br>◯ 7 days<br>◯ 10 days<br>◯ 14 days<br>◯ > 14 days

11. A 40-year-old female with presents fever, cough and diagnosed with pneumonia and bacteremia due to S. pneumoniae. She was treated with IV ceftriaxone and improved clinically.

Would you complete this patient's antibiotic course with ORAL therapy?	◯ Yes (always)<br>◯ No (never)<br>◯ Depends (Only if no persistent fever)

Would you routinely repeat blood cultures to confirm clearance of bacteremia?	◯ Yes<br>◯ No
How long would you treat this patient's bacteremia?	◯ < 7 days<br>◯ 7 days<br>◯ 10 days<br>◯ 14 days<br>◯ > 14 days

12. A 60-year-old female with presents fever with methicillin-susceptible S. aureus bacteremia, due to a left knee septic arthritis and had adequate surgical washout. She was treated with IV cefazolin and improved clinically.

Would you complete this patient's antibiotic course with ORAL therapy?	◯ Yes (always)<br>◯ No (never)<br>◯ Depends (Only if no persistent fever)
Would you routinely repeat blood cultures to confirm clearance of bacteremia?	◯ Yes<br>◯ No
How long would you treat this patient's bacteremia?	◯ < 7 days<br>◯ 7 days<br>◯ 10 days<br>◯ 14 days<br>◯ > 14 days

13. A 26-year-old female presents with fever and bacteremia with Prevotella oralis (gram-negative anaerobic rod), later found to have a ruptured appendicitis. She underwent laparoscopic appendectomy, and was treated with IV piperacillin/tazobactam initially. She improved clinically on this therapy.

Would you complete this patient's antibiotic course with ORAL therapy?	◯ Yes (always)<br>◯ No (never)<br>◯ Depends (Only if no persistent fever)
Would you routinely repeat blood cultures to confirm clearance of bacteremia?	◯ Yes<br>◯ No
How long would you treat this patient's bacteremia?	◯ < 7 days<br>◯ 7 days<br>◯ 10 days<br>◯ 14 days<br>◯ > 14 days

< Demographics >

Please indicate the country where you currently practice	__________________________________
a. Current Position	◯ Physician - resident<br>◯ Physician - fellow<br>◯ Attending Physician - 0-5 yrs in practice<br>◯ Attending physician - 6-10 yrs in practice<br>◯ Attending physician - 11-24 yrs in practice<br>◯ Attending physician - >25 yrs in practice<br>◯ Advanced practice provider<br>◯ Pharmacist - In training<br>◯ Pharmacist - In practice

b. Age	◯ < 30 yrs<br>◯ 30-40 yrs<br>◯ 41-50 yrs<br>◯ >50 yrs<br>◯ Choose not to identify
c. Gender	◯ Female<br>◯ Male<br>◯ Transgender Male<br>◯ Transgender Female<br>◯ Other gender not listed above<br>◯ Choose not to identify
d. Medical specialty	◯ Family medicine<br>◯ General internal medicine<br>◯ Infectious diseases (adult or pediatrics)<br>◯ Other internal medicine subspecialty<br>◯ Pediatrics or pediatric subspecialties<br>◯ Surgery or surgical subspecialties<br>◯ Other specialty not listed
